# miR-205-5p Downregulation and *ZEB1* Upregulation Characterize the Disseminated Tumor Cells in Patients with Invasive Ductal Breast Cancer

**DOI:** 10.3390/ijms23010103

**Published:** 2021-12-22

**Authors:** Lenka Kalinkova, Nataliia Nikolaieva, Bozena Smolkova, Sona Ciernikova, Karol Kajo, Vladimir Bella, Viera Horvathova Kajabova, Helena Kosnacova, Gabriel Minarik, Ivana Fridrichova

**Affiliations:** 1 Department of Genetics, Cancer Research Institute, Biomedical Research Center of the Slovak Academy of Sciences, 84505 Bratislava, Slovakia; lenka.kalinkova@savba.sk (L.K.); nataliia.nikolaieva@savba.sk (N.N.); sona.ciernikova@savba.sk (S.C.); karol.kajo@ousa.sk (K.K.); helena.svobodova@savba.sk (H.K.); 2 Department of Molecular Oncology, Cancer Research Institute, Biomedical Research Center of the Slovak Academy of Sciences, 84505 Bratislava, Slovakia; bozena.smolkova@savba.sk (B.S.); viera.kajabova@savba.sk (V.H.K.); 3 Department of Pathology, St. Elisabeth Cancer Institute, 81250 Bratislava, Slovakia; 4 Department of Senology, St. Elisabeth Cancer Institute, 81250 Bratislava, Slovakia; vladimir.bella@ousa.sk; 5 Institute of Molecular Biomedicine, Faculty of Medicine, Comenius University, 81108 Bratislava, Slovakia; gabriel.minarik@gmail.com

**Keywords:** invasive ductal breast cancer, *CDH1* gene, EMT genes, miRNA and mRNA expression, E-cadherin

## Abstract

Background: Dissemination of breast cancer (BC) cells through the hematogenous or lymphogenous vessels leads to metastatic disease in one-third of BC patients. Therefore, we investigated the new prognostic features for invasion and metastasis. Methods: We evaluated the expression of miRNAs and epithelial-to-mesenchymal transition (EMT) genes in relation to *CDH1*/E-cadherin changes in samples from 31 patients with invasive ductal BC including tumor centrum (TU-C), tumor invasive front (TU-IF), lymph node metastasis (LNM), and CD45-depleted blood (CD45-DB). Expression of miRNA and mRNA was quantified by RT-PCR arrays and associations with clinico-pathological characteristics were statistically evaluated by univariate and multivariate analysis. Results: We did not verify *CDH1* regulating associations previously described in cell lines. However, we did detect extremely high *ZEB1* expression in LNMs from patients with distant metastasis, but without regulation by miR-205-5p. Considering the ZEB1 functions, this overexpression indicates enhancement of metastatic potential of lymphogenously disseminated BC cells. In CD45-DB samples, downregulated miR-205-5p was found in those expressing epithelial and/or mesenchymal markers (CTC+) that could contribute to insusceptibility and survival of hematogenously disseminated BC cells mediated by increased expression of several targets including *ZEB1*. Conclusions: miR-205-5p and potentially *ZEB1* gene are promising candidates for markers of metastatic potential in ductal BC.

## 1. Introduction

The International Agency for Research on Cancer’s GLOBOCAN 2018 reported the global burden of cancer across 20 world regions and revealed almost 2.1 million new breast cancer (BC) patients and 0.63 million associated deaths. That means a 24.2% incidence and 15% mortality rate in female cancer patients [1]. Among BC patients, metastatic disease has been reported in the range from 7% to 35% [2,3,4]. Metastatic breast cancer is generally considered incurable and regardless of some improvement in overall survival only 13% of BC patients with primary stage IV survive 10 years after diagnosis [5]. Cancer cells spread from the tumor mass either via blood or via lymphatic circulation after the intensive neo-vascularization and neo-lymphangiogenesis, respectively [6]. Detached cancer cells enter blood vessels directly, but those traveling through the lymphatic vasculature either form metastases in lymph nodes or they pass into the blood circulation through the thoracic duct [7]. However, the factors determining the method of cancer cell dissemination depend mainly on the cancer type and features of the tumor microenvironment [8].

In BC, lymphovascular invasion inside and around the tumor tissue indicate the mechanisms of cancer spread and metastases in lymph nodes are considered as the key prognostic marker of tumor spread and aggressiveness [9,10,11]. On the other hand, many studies performed in the last decade have investigated the role of circulating tumor cells (CTCs) in haematogenous dissemination of cancer and their clinical utility in prognosis is under examination in ongoing clinical trials. Detection and count of CTCs are used as independent prognostic factors for primary and metastatic BC contributing to the monitoring and treatment stratification of BC patients [12,13,14]. Various technologies for CTC detection and isolation have been developed (including the validated CellSearch^®^ system) and most of them have utilized the epithelial characters for CTC enrichment [15,16]. In BC, heterogeneous CTC subpopulations were found, counting cells with epithelial characters co-existing with those with epithelial-to-mesenchymal transition (EMT) features; however, EMT CTCs have been identified more frequently in metastatic BC patients and are associated with poor prognosis [17,18,19,20].

Mesenchymal cell phenotype is associated with an increased migratory capacity, invasiveness, apoptosis, and resistance; therefore, EMT is considered an essential event in BC haematogenous dissemination [20,21]. On the other hand, TGF-1-induced EMT was recently described to promote the chemotaxis-mediated migration of BC cells through the lymphatic vessels [22]. The main feature of EMT induction is the loss of cell–cell adherent junctions via inhibition of E-cadherin encoded by the *CDH1* gene. Key EMT inducers, which act as direct *CDH1* repressors, belong to three distinct families; the Snail family (SNAIL1, SNAIL2/SLUG, and SNAIL3/SMUC), the Zeb family (ZEB1/2), and the b-HLH family (TWIST1/2). Their encoding genes inhibit E-cadherin expression via binding to the E-box elements in the promoter region of the *CDH1* gene [23,24].

Genetic and epigenetic mechanisms of E-cadherin downregulation were previously described. Several *CDH1* somatic or germline mutations and loss of heterozygosity were found almost exclusively in invasive lobular BC [25,26]. In addition, epigenetic modulation by *CDH1* promoter methylation [27,28] or by miRNA post-transcription regulation have been documented [29].

In BC cells, MYC/MYCN-activated miR-9 was found to be a direct regulator of the *CDH1* gene, and increased miR-9 levels were associated with metastatic status and local recurrence in BC patients [30,31]. The miR-221 a member of the miR-221/222 cluster directly targeted the open reading frame of *CDH1* and a higher miR-221 expression, significantly upregulated by SNAIL2/SLUG, was observed in metastatic BC cells and BC patients with LNMs and distant metastases [32,33,34]. Moreover, the miR-221/222 cluster decreased expression of E-cadherin indirectly via targeting of transcription repressor *TRPS1*, the direct regulator of *ZEB2* transcription, resulting in abundance of *ZEB2* and promotion of EMT [35].

Several *CDH1* repressors and therefore EMT inducers were found to be regulated by members of a well-investigated miR-200 cluster including miR-200b/200a/429 and miR-200c/141. Decreased expression of all members of the miR-200 cluster followed by upregulation of *SNAI1*, *SNAI2*, *ZEB1*, and *ZEB2* genes was observed in EMT in vitro models of breast basal cell lines and BC patients, more markedly in those with metaplastic tumors. Moreover, the inactivation of miR-200c/141 expression could be caused by hypermethylation of its promoter [36]. Among them, miR-200c targeted *ZEB1* and the other two *TKS5* and *MYLK* genes acting in invadopodia formation. Co-expression of these three genes and low expression of miR-200c in several BC lines as well as in BC patients are associated with EMT activation, higher invasion and invadopodia creation [37,38]. In a recent study, miR-200c targeted *ZEB2* and the role of this repressor in metastasis was found in triple-negative BC (TNBC) cells and tissues [39]. Furthermore, both *ZEB1* and *ZEB2* were directly regulated by miR-205 and decreased levels of miR-205 initiated EMT and were associated with a metastatic phenotype of BC patients [40,41].

Other BC in vitro studies showed that expression levels of miR-203 and miR-200 cluster were decreasing in a time-dependent manner during SNAI1-induced EMT. The miR-203-reduced *SNAI1* endogenous levels generated a double-negative miR-203/SNAI1 feedback loop and together with the miR200/ZEB1 feedback loop, the plasticity of the epithelial cell during differentiation and tumorigenesis was controlled [42]. The miR-203 also directly regulated *SNAI2* gene and this miRNA was upregulated and downregulated in non-metastatic and metastatic cell lines, respectively, compared to non-tumorigenic cells. Moreover, decreasing levels of miR-203 in metastatic cells were associated with promoter hypermethylation [43]. Downregulation of miR-124 was found in TNBC cell lines and patient tissues. It was documented that miR-124 directly targeted the *ZEB2* gene and contributed to EMT and metastasis suppression [44]. Other authors showed that miR-124 also directly targeted the *SNAI2/SLUG* gene, which allowed E-cadherin expression and inhibition of cell invasion and metastasis. In BCs, the significantly reduced miR-124 levels negatively correlated with histological grade [45].

Compared to the above-mentioned EMT-associated transcription factors, the miRNA regulation of *TWIST1* expression in BC has been less frequently investigated. The *TWIST1* gene was directly targeted by miR-720 and significant downregulation of miR-720 followed by increasing *TWIST1* levels were observed predominantly in metastatic BC [46].

Generally, in BCs and many other epithelial cancers, the attenuation of E-cadherin adhesion is considered the main event in invasion and metastasis. Regarding the prognostic value, high levels of E-cadherin were found to be a good prognostic marker in most cancers. In a previous study, the variable trend of decreasing E-cadherin expression was observed from ductal BCs in situ, from ductal BCs without LNMs to those with LNMs, but increased E-cadherin levels were found in LNM tissues compared to primary tumors [47]. However, a more recent study showed that high E-cadherin correlated with shorter survival in invasive ductal BCs in contrast to the lobular subtype of BC, and reduced or lost E-cadherin expression was inversely associated with tumor stage, indicating more complex and possible divergent functions of this protein in BCs [48]. This hypothesis is supported by results from mouse experimental models, where the loss of E-cadherin improved cancer cell invasion, but reduced cell proliferation and survival, the number of CTCs in systemic circulation, and dissemination of cancer cells in distant organs. The authors indicated that E-cadherin could contribute to metastasis through apoptosis inhibition [49].

**Figure 1:**
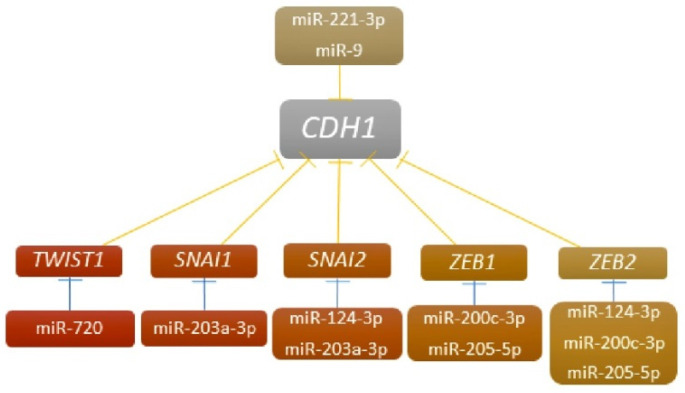
Diagram of associations in inhibition of *CDH1* expression adapted from results of in vitro studies.

In this study, we investigated epigenetic changes associated with regulation of the E-cadherin encoding *CDH1* gene to contribute to a better understanding of the specific functions of E-cadherin and associated miRNAs and genes in invasive ductal BCs. We analyzed the expression of *CDH1*, five well-known EMT genes, and seven regulating miRNAs (Figure 1) in tumor centrum (TU-C), tumor invasive front (TU-IF), and in lymph node metastasis (LNM) and CTC-enriched blood fraction samples (by CD45 depletion) to identify aberrantly expressed miRNAs and genes, and understand their associations with clinical parameters of invasive and metastatic processes including LNM, CTC, lymphovascular invasion (LVI), and distant metastasis (MTS). Our results did not confirm *CDH1* regulating associations previously described in cell line models. However, we detected extremely high *ZEB1* expression in LMN samples from patients with MTS, which was not regulated by miR-205-5p. Due to *ZEB1* functions, its overexpression indicates enhancement of metastatic potential of disseminated BC cells spread through the lymphatic vessels. In CD45-depleted blood (CD45-DB) fractions, the downregulated miR-205-5p was found in samples expressing epithelial and/or mesenchymal markers (CTC+) that could contribute to reduced susceptibility and increased survival of hematogenously disseminated BC cells mediated by increased expression of several target genes including *ZEB1*. Both, miR-205-5p and potentially *ZEB1* are promising candidates as markers for metastatic potential of disseminated ductal BC cells.

## 2. Results

### 2.1. miRNA and mRNA Expressions vs. Controls

**Figure 2:**
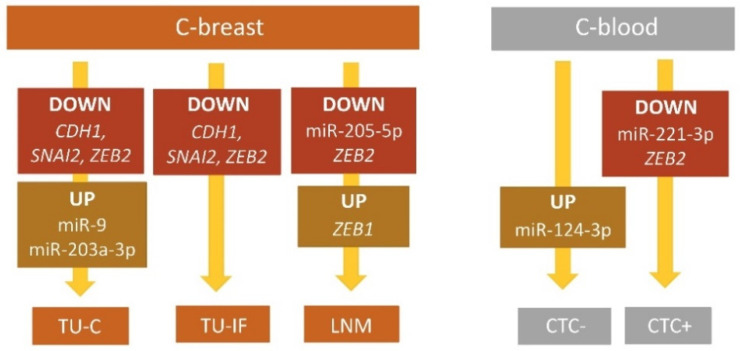
Significantly different expressions of miRNAs and mRNA of *CDH1* and EMT genes in tumor centrum (TU-C), tumor invasive front (TU-IF) and lymph node metastasis (LNM), and in CD45-DB fractions enriched by CTCs compared to adequate controls (C-breast, non-neoplastic breast tissues and C-blood, CD45-depleted fractions from healthy women).

In the group of 31 patients with invasive ductal breast cancer, we evaluated the expressions of seven miRNAs regulating *CDH1* and EMT repressors (miR-9-5p, miR-124-3p, miR-200c-3p, miR-203a-3p, miR-205-5p, miR-221-3p, and miR-720) and mRNA expression for *CDH1* and associated EMT genes (*TWIST1*, *SNAI1*, *SNAI2*, *ZEB1*, and *ZEB2*). The analyses were performed in TU-C, TU-IF and LNM samples, and in CD45-DB fractions enriched by CTCs with and without identified epithelial and/or mesenchymal markers (CTC+ and CTC-, respectively). The controls for all patients’ tissue samples and depleted fractions were non-neoplastic breast specimens (C-breast) and CD45-depleted fractions from healthy women (C-blood). In TU-C, significant upregulation was detected for miR-9-5p (fold change, FC 7.915, *p* = 0.042) and miR-203a-3p (FC 2.356, *p* = 0.042) and downregulation for *CDH1* (fold change, FC 0.123, *p* = 0.002), *SNAI2* (FC 0.16, *p* = 0.021) and *ZEB2* (FC 0.125, *p <* 0.001) genes compared to controls. Similarly, TU-IFs were downregulated for *CDH1* (FC 0.108, *p* = 0.001), *SNAI2* (FC 0.102, *p* = 0.002) and *ZEB2* (FC 0.086, *p <* 0.001) genes and LNM samples were downregulated for miR-205-5p (FC 0.21, *p* = 0.012), and upregulated for *ZEB1* (FC 22.08, *p* = 0.043) and *ZEB2* (FC 0.122, *p* = 0.003). In CTC-depleted bloods, miR-124-3p (FC 9.766, *p* = 0.036) was upregulated and in CTC+ samples, miR-221-3p (FC 0.289, *p* = 0.017) and *ZEB2* (FC 0.395, *p* = 0.037) were downregulated compared to controls (Figure 2, Table 1).

### 2.2. miRNA and mRNA Expressions in Different Types of Samples

**Figure 3:**
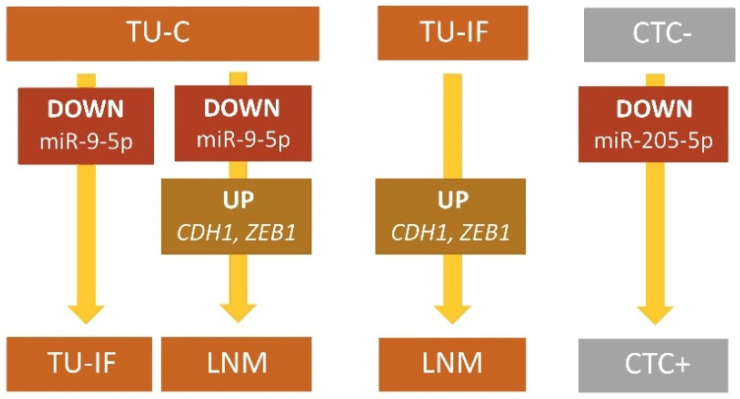
Expression of miRNAs, and *CDH1* and EMT genes between different types of BC patients’ samples, namely in tumor centrum (TU-C), tumor invasive front (TU-IF) and lymph node metastasis (LNM), and in CD45-depleted blood (CD45-DB) fractions without and with identified epithelial and/or mesenchymal markers (CTC- and CTC+).

Expression of two miRNAs, and *CDH1* and *ZEB1* genes was found to be statistically different between BC samples. In both TU-IF and LMN, miR-9-5p was downregulated and in LNM, *CDH1* and *ZEB1* were upregulated compared to TU-C. Similarly, upregulated *CDH1* and *ZEB1* genes were found in LNM against expressions in TU-IF. CTC+ samples presented miR-205-5p downregulation compared to CTC- (Table 1, Figure 3).

To evaluate the inhibitory function of miRNAs we analyzed the correlation between expressions of miRNAs, and *CDH1* and EMT genes, and between *CDH1* and each of EMT genes (Table 2 and Table S1). Negative correlation was found between miR-221-3p and *TWIST1* gene in TU-Cs (correlation coefficient (r), r = −0.470, *p* = 0.015), and miR-9 and *SNAI1* gene in LNMs (r = −0.607, *p* = 0.013). In CD45-DB fractions the negative correlations were observed more frequently, in CTC- samples between miR-124-3p and *TWIST1* (r = −0.883, *p* = 0.020), and miR-221-3p and *ZEB2* (r = −0.543, *p* = 0.024). In CTC+ specimens, miR-9, miR-205-5p and miR-720 negatively correlated with both *SNAI1* and *ZEB1* (r ranged from −0.853 to −0.588 and *p*-value from 0.001 to 0.044), miR-221-3p with *ZEB1* (r = −0.610, *p* = 0.035) (Table 2). No negative correlations were detected between expressions of *CDH1* and any of EMT genes in tumor, LNM and CTC samples (Table S1).

In this study, several associations between miRNAs and genes indicating regulation events previously documented by in vitro results were shown. In TU-Cs, upregulated miR-9 and downregulated *CDH1*, and upregulated miR-203a-3p, and downregulated *SNAI2* were found compared to controls. LNM samples presented downregulation of miR-205-5p with upregulation of the *ZEB1* gene (Table 1). However, in none of these associations were significant negative correlations between miRNA and mRNA expressions of *CDH1* and EMT genes observed.

### 2.3. Association between miRNA and mRNA Expression and Clinico-Pathological Parameters

**Figure 4:**
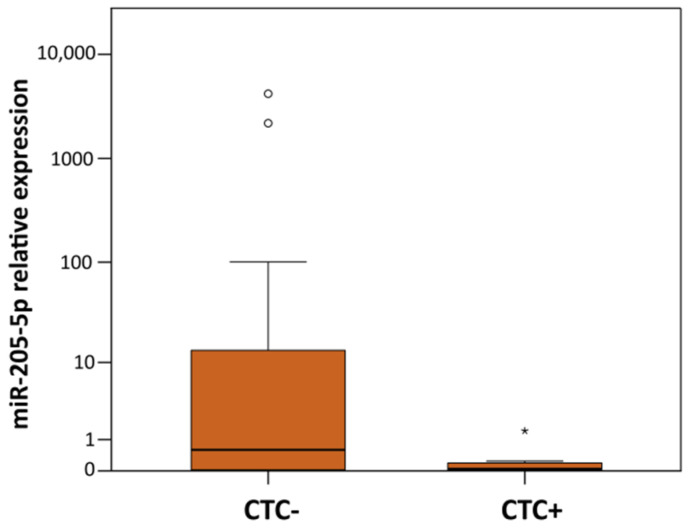
Individual expression levels of miR-205-5p in CD45-depleted peripheral blood of CTC negative (CTC-) and positive (CTC+) BC patients. The median is depicted by a horizontal line within each bar. The length of the boxes is the interquartile range (IQR) that represents values between the 75th and 25th percentiles of individual fold change values. Values more than 3 IQRs from the end of the box are labeled as extreme (*). Values more than 1.5 IQRs but less than 3 IQRs from the end of the box are labeled as outliers (O). Abbreviations: BC, breast cancer; CTC, circulating tumor cells.

Using univariate statistical analysis, we found spectrum of associations between up- or downregulated expressions of miRNAs and *CDH1* and EMT genes and relevant clinico-pathological characteristics for each group of samples (Table 3). In TU-C, downregulated levels of miR-124-3p and miR-203a-3p were detected in patients with MTS and TNM staging III and IV compared to those without MTS and lower TNM, respectively. Furthermore, miR-200c-3p was downregulated in HER2 positive BCs and reduced *CDH1*, *SNAI1*, and *ZEB2* expression was identified in ER+ and /or PR+ tumors. TU-IF samples showed downregulation for miR-200c-3p in ER+ tumors, decreased expression for *TWIST1* in ER+ BCs and several combinations of downregulated EMT genes, namely *SNAI2* and *ZEB1* associated with lymph node metastasis (LNM) positive phenotype and higher TNM, respectively; *SNAI2* and *ZEB2* with distant metastasis (MTS) presence and *SNAI2*, and both, *ZEB1* and *ZEB2* with LVI. Decreased *ZEB2* was also associated with tumors sized ≤20 mm. In LNM tissues, upregulation of miR-124-3p, *SNAI1* and *ZEB1* was associated with ER+, higher TNM and MTS+ in the primary tumor, respectively. In CD45-DB samples, downregulation of *SNAI1* and upregulation of miR-9 were found in patients with HER2+ tumors, and upregulation of *CDH1* in grade 3 cancers, respectively. miR-205-5p were found upregulated in patients with LNM+ and higher TNM but significantly downregulated in patients with CTC+ phenotype (Figure 4). FCs and *p*-values for these associations are summarized in Table 3.

Immunohistochemical analyses in TU-C samples showed that 17 and 5 patients presented strong homogenous expression (3+) and heterogeneous strong and moderate expression (3+ and 2+) of E-cadherin in different portions in individual patients, respectively. These samples were considered as those with high E-cadherin expression. In nine patients with low E-cadherin expression, strictly heterogeneous phenotype with different portion of strong, moderate, poor, and no expression (3+/2+/1+, 3+/2+/1+/0 and 2+/1+) was observed. The associations between *CDH1* gene expression in high and low E-cadherin expression were tested by univariate statistical analysis. An increasing trend in *CDH1* expression in E-cadherin high compared to low expression was found; however, without upregulation and statistically significant difference in *CDH1* expression between E-cadherin high and low groups (FC 1.134, *p* = 0.777).

Multivariate analysis was used to test for risk factors influencing hematogenous and lymphogenous dissemination, namely CTC, LNM, and LVI positivity, and the presence of distant MTS. Variables that were significant in univariate analysis were used in multivariate analysis.

Firstly, logistic regression was applied to predict the potential influence of miR-205-5p expression on the dissemination of tumor cells characterized by CTC positivity. In multivariate models, the clinical parameters of age, tumor size, histological grade, and HER2 status were included to control for potential confounders. The multivariate model correctly classified 88.9% of CTC negative and 91.7% of CTC positive patients, respectively, with an overall success rate of 90.0%. The presence of CTC positivity was negatively correlated with miR-205-5p expression in CD45-DB fractions (Table 4). Multivariate models for LNM, LVI, and MTS risk prediction were not significant.

## 3. Discussion

Based on the generally accepted knowledge of the key role of E-cadherin (encoded by *CDH1* gene) in cancer cell spread, we investigated the influence of aberrant expression of several EMT genes and their regulating miRNAs, in addition to miRNAs which directly targeted *CDH1* gene expression on ductal BC development in several stages of disease.

Our results showed decreasing levels of *CDH1* expression in both TU-C and TU-IF compared to C-breast, but without a statistically significant difference between the two. LMN samples were characterized by a similar expression of *CDH1* gene as in C-breast, but its upregulation compared to TU-C and TU-IF indicates a possible role of *CDH1* gene in lymphogenous cancer spread. On the other hand, similar *CDH1* expression was found in CTC- and CTC+ samples compared to C-blood.

In our patients, immunohistochemical E-cadherin expression was carefully evaluated in TU-C. In all tumors, full E-cadherin expression (3+) was found in 17 samples; however, 14 presented locally decreased levels of E-cadherin. Among them, increasing *CDH1* levels were observed in those with high E-cadherin (3+ and 3+/2+ phenotype) compared to low levels (3+/2+/1+, 3+/2+/1+/0 and 2+/1+), but without a statistically significant difference. The reason we were not able to show *CDH1*/E-cadherin association could the variability of immunohistochemistry results in ductal BCs. In technical terms, it may be that qualitative analyses of *CDH1* expression and semiquantitative E-cadherin immunohistochemistry cannot be performed in the same region of tumor. However, the association between expression of *CDH1* and E-cadherin has been documented by other authors [50] and the upregulated *CDH1* levels in LNM tissues identified in our study could correspond with the previously published increase in E-cadherin expression in LNM tissues [47].

In this study, the relatively complicated scheme of *CDH1* regulation by miRNAs and EMT genes was used to investigate possible regulators of *CDH1* in several types of samples from invasive ductal BC patients. All evaluated associations were previously identified in vitro [30,33,38,40,42,43,44,45,46]. Among them, only upregulated miR-9 with downregulated *CDH1* associating with invasive phenotype and upregulated miR-203a-3p with downregulated *SNAI2* gene indicating inactive EMT process were detected in TU-C. Our results were in accordance with other studies showing upregulation of miR-203a-3p in cell lines and primary BCs. According to the findings, a negative association between downregulated miR-203a-3p and upregulated SNAI2 was observed in metastatic cells [43,51,52]. LNM samples presented downregulated levels of miR-205-5p with upregulation of the *ZEB1* gene, indicating the important role of *ZEB1* in invasion. However, a negative correlation between the expression of these miRNAs and associated genes was not detected. On the other hand, we found several negative correlations that were not investigated in vitro. They could designate the new regulating connection as in the case of miR-9 and *SNAI1* expression observed in both, LNM and CTC+ samples. The other possibility is that these findings show only the independent co-existence observed in particular stages of BCs in relation to their functions (Table 2).

Similarly to LNM tissues, CTC+ samples showed miR-205-5p downregulation, and a negative correlation between miR-205-5p and *ZEB1* expression was detected.

The ZEB1 transcription factor is regulated by multiple signaling pathways and molecules as TGF-β, β-catenin and miRNAs, and it alone regulates a high number of genes, as was found in TNBCs [53,54]. In addition to EMT promotion, *ZEB1* overexpression contributes to maintenance of stem-like features, immune evasion, and epigenetic reprogramming [55]. Moreover, *ZEB1* initiates chemoresistance but inhibits the apical–basal polarity of cancer cells and antiestrogen sensitivity [53]. All these functions could contribute to the insusceptibility and survival of disseminated BC cells located in LNM samples due to elevated levels of the *ZEB1* gene found in our study.

To evaluate the association between aberrant expression of *CDH1*, and regulation of miRNAs and EMT genes and selected clinico–pathological parameters, we found a varying combination of changes in individual types of patient’s samples. Patients with ER+ tumors showed downregulation of *SNAI1* and *TWIST1* in TU-C and TU-IF, respectively. Similarly, downregulation on protein and mRNA levels were observed in primary BCs by other authors, respectively [56,57]. In our study, upregulation of miR-200c-3p and miR-124-3p in TU-IF and LNM samples was observed in ER+ patients compared to ER-, respectively. The identical finding for miR-200c-3p was described also in other studies [58,59]. Differences in PR status were observed only in TU-C where PR+ samples were characterized by downregulated *CDH1* and *SNAI1* genes. Other authors identified a similar association between decreased SNAI1 protein and the PR+ phenotype [60]. In addition, HER2+ against HER2- tumors showed downregulation miR-200c-3p, and upregulation of miR-9 and downregulation of *SNAI1* were found in TU-C and CD45-DB fractions, respectively. In patients with advanced BCs in advanced stage (TNM III and IV), downregulated miR-203a-3p, and *SNAI2* with *ZEB1* were detected in TU-C and TU-IF, and upregulation of *SNAI1* and miR-205-5p was found in samples with disseminated cancer cells, LNM and CD45-DB fractions, respectively. Finally, in TU-F, tumors >20 mm presented downregulated *ZEB2* and in CD45-DB fractions, upregulation of *CDH1* in Grade 3 tumors compared to patients with smaller and highly or moderately differentiated BCs, respectively.

For evaluation of the influence of expression change on hematogenous or lymphogenous dissemination, the presence of LVI, LNM, CTC, and MTS were crucial parameters that were consequently used for the creation of multivariate models. In patients with LVI+, downregulation of *SNAI2*, *ZEB1*, and *ZEB2* was observed in TU-IF. The presence of LNM was associated with downregulation of *SNAI2* and *ZEB1* in TU-IF and upregulation of miR-205-5p in the CD45-DB fraction. Patients with distant metastasis showed downregulated miR-124-3p in TU-C, and *SNAI2* and *ZEB2* in TU-IF. Consistent with these findings, in vitro and in vivo studies showed that miR-124-3p inhibit the metastasis process [61,62]. The markedly upregulated levels of *ZEB1* in LMN tissues from patients with MTS could indicate its previously hypothesized role in metastasis [63]. The presence of CTC was associated with downregulation of miR-205-5p in the CD45-DB fractions that was verified in the multivariate risk model for CTC risk prediction (Table 4).

Regardless of the many questions remaining about the role of miR-205-5p in normal breast physiology, tumor-suppressor activities of this miRNA were documented in many studies. Decreasing levels of miR-205-5p were observed from less aggressive BC subtypes and ER+/PR+ tumors to more aggressive cases as TNBCs and those with high metastatic capabilities, poor response to therapy and patient survival [41,64]. To date, more than 20 genes targeted by miR-205-5p associating with processes and pathways involved in breast tumorigenesis were identified [64,65]. Decreased levels of miR-205-5p expression in CTC+ samples allow higher expression of the *ZEB1* gene, which could contribute to better condition and protection of cancer cells by several processes as previously discussed. Expression of other target genes *ITGA5* and *NOTCH2* could improve the stemness and metastatic potential of hematogenously disseminated cancer cells [66,67]. Moreover, after the reduction in miR-205-5p, CTCs could acquire chemoresistance features resulting from overexpression of *VEGF-A* and *FGF2*, leading to increased apoptosis upon chemotherapy treatment [68].

To our knowledge, miRNA expression analyses in CD45-DBs have been published very rarely. We found only one in silico study, in which specific differentially expressed miRNAs were identified, miR-99a and miR-151-3p for ductal BCs in situ, miR-145 and miR-210 for invasive BCs, and miR-361-5p and miR-205 for metastatic BCs [41]. Our study therefore brings original results. On the other hand, gene expression profiles were investigated in CTC samples by several research groups. In these studies, the gene expression profiles in CTCs obtained from patients with metastatic BCs were different compared to primary tumors that can be utilized for characterization of CTCs, and evaluation of prognosis and therapeutic prediction. However, expression profiles of mesenchymal CTCs were omitted for EpCAM separation of CTC-enriched fractions [69,70,71,72]. In our study, CTC+ samples were characterized by epithelial and/or mesenchymal features; therefore, we consider our results to be more objective.

Generally, the positive expression of E-cadherin is used to discriminate between ductal and lobular subtypes of BC. However, detailed examination reveals different levels of E-cadherin inhibition in the many regions of invasive ductal tumor tissues. In this BC subtype, genetic changes in the E-cadherin encoding gene *CDH1* are very rare; therefore, we investigated the influence of aberrant expression of *CDH1* and regulating miRNA and EMT genes on invasive and metastatic features in samples which represent several stages of BC cell dissemination. In this study, we showed a variable spectrum of upregulated or downregulated expressions of the *CDH1* gene and associated miRNAs and EMT genes and did not verify any regulating relationships, which were previously described in cell line studies, except an association between miR-205-5p and *ZEB1* expressions in the CTC+ fraction. However, we did observe extremely high *ZEB1* expression in LMN samples obtained from patients with distant metastases that was not explained by miR-205-5p decreasing. This finding indicates that *ZEB1* overexpression could enhance the metastatic potential of cancer cells disseminated through the lymphatic circulation. In CD45-DB fractions, the samples with the identified presence of CTCs showed downregulation of miR-205-5p expression that could contribute to maintaining the stemness and initiation of such protective features as immune evasion and chemoresistance through the increased expression of several target genes including *ZEB1*. Together, we identified miR-205-5p and *ZEB1* as potential markers for metastatic behavior of disseminated BC cells originating from a ductal tumor; however, their clinical relevance needs to be widely investigated.

## 4. Materials and Methods

### 4.1. Patients

We analyzed patient’s RNA samples isolated from CD45-DB fractions and FFPE specimens from the central region and invasive front of tumor and lymph node metastases. The controls were non-neoplastic breast tissues and CD45-DB fractions of age-matched women. At the Department of Senology and Department of Pathology, St. Elisabeth Cancer Institute, Bratislava, 69 patients suspected of an invasive type of breast cancer were pre-selected and blood samples were collected. After the evaluation of post-operation tumor samples, 31 patients with invasive ductal BC were included in this study. 13 non-neoplastic breast tissues and 12 CD45-DB from heathy women at matched age were used as controls. This study was approved by St. Elizabeth Cancer Institute Review Board in Bratislava and written informed consent was obtained from all patients and controls. The age of patients ranged from 42 to 86 years, (median 65 years), controls were aged between 52 and 79 years (median 67 years) and between 54 and 66 years (median 59 years) in breast tissue and CD45-DB samples, respectively. No statistical differences in age were found between patients and controls. The clinical and histopathological characteristics and immunohistochemical data (tumor size, histological grade, LN and MTS status, TNM stage, LVI, hormone receptor (ER, PR) and HER2 status, Ki-67 and E-cadherin expression) were obtained from patients records and tumors were defined according to TNM classification (Table 5). No patient underwent preoperative radiotherapy or chemotherapy before specimen collection, and control women had no signs or symptoms of cancer or other serious diseases.

### 4.2. CD45 Depletion of Peripheral Blood and CTC Detection

Preparation of CD45-negative blood fractions was performed by RosetteSep Human CD45 Depletion Cocktail (StemCell Technologies, Vancouver, BC, Canada) based on depletion of CD45+ peripheral blood cells. Quantitative real-time polymerase chain reaction (qRT-PCR) was used for CTCs detection in CD45-DB samples as has been previously described [73,74]. RNA extraction from CD45-DB fractions was exposed to detection of EMT-inducing transcription factors gene transcripts (*TWIST1*, *SNAIL1*, *SLUG* and *ZEB1*) and epithelial antigen (*CK19*) by TaqMan assays (LifeTechnologies, Carlsbad, CA, USA). The higher expression levels of either epithelial and/or mesenchymal gene transcripts than those of healthy donors were considered as CTCs positive.

### 4.3. miRNA and mRNA Isolation and Real-Time PCR

For gene expression analyses, miRNA and mRNA from CD45-DB fraction and FFPE breast tissues were used. miRNAs from CD45-DB fraction were isolated using the miRNeasy Mini Kit (Qiagen, Hilden, Germany) and miRNAs from FFPE breast tissues were isolated using the miRNeasy FFPE Kit (Qiagen, Hilden, Germany) according to the manufacturer’s instructions.

mRNAs from CD45-DB fractions were isolated using the miRNeasy Mini Kit–RNeasy MinElute Cleanup Kit (Qiagen, Hilden, Germany) and mRNAs from FFPE samples were extracted using the PureLink FFPE Total RNA Isolation Kit following the provided protocol (Invitrogen Corporation, Carlsbad, CA, USA). miRNAs and mRNA samples were reversely transcribed into cDNA using the miScript II RT Kit (Qiagen, Hilden, Germany) and RevertAid First Strand cDNA Synthesis Kit (Thermo Fisher Scientific, Vilnius, Lithuania), respectively.

For real-time polymerase chain reaction (RT-PCR) Custom miScript miRNA PCR Array (CMIHS02741, Qiagen, Germany) was used. For expression analyses of mature forms of hsa-miR-9-5p, hsa-miR-124-3p, hsa-miR-203a-3p, hsa-miR-200c-3p, hsa-miR-205-5p, hsa-miR-221-3p, and hsa-miR-720, the miScript SYBR Green PCR Kit (Qiagen, Germantown, MD, USA) was used. Reactions were performed in AriaMx Real-Time PCR System (Agilent, Santa Clara, CA, USA) using the following conditions: pre-denaturation at 95 °C for 15 min, followed by 40 cycles at 94 °C 15 s, 55 °C for 30 s, and 70 °C for 30 s, followed by melt cycle at 95 °C for 30 s, 65 °C for 30 s, and 95 °C for 30 s. Among three reference controls (Snord61, Snord72, and Snord95), Snord95, with the most stable expression, was selected for normalization of Ct values.

qRT- PCR detection and expression of *CDH1*, *TWIST1*, *SNAI1*, *SNAI2*, *ZEB1*, *ZEB2* and *18S* were performed using TaqMan Gene Expression Assays—single tube assays (Thermo Fisher Scientific, Pleasanton, CA, USA): *CDH1*–Hs01013959_m1, *TWIST1*–Hs00361186_m1, *SNAI1*–Hs00195591_m1, *SNAI2*–Hs00161904_m1, *ZEB1*–Hs01566408_m1, *ZEB2*–Hs002007691_m1, 18S–Hs_9999991_s1. qRT-PCR reactions were carried out in an AriaMx Real-Time PCR System (Agilent, Santa Clara, CA, USA) at following settings: uracil-N-glycosylase incubation 1 cycle at 50 °C for 2 min, enzyme activation 1 cycle at 95 °C for 20 s, 40 cycles at 95 °C for 30 s denaturation and 60 °C for 30 s annealing. For all fluorescence-based RT-PCR, fluorescence was detected between 10 and 40 cycles for the reference (18 S) and target genes. Fold change was calculated as normalized relative gene expression using formula 2^−ΔΔCt^.

### 4.4. Immunohistochemical Analyses of E-Cadherin

Immunohistochemistry for detection of E-cadherin was performed on paraffin sections with ready to use reagents using an automated immunostainer, Autostainer Link 48 (Dako; Agilent Technologies, Inc., Santa Clara, CA, USA). Primary E-cadherin antibody (FLEX Monoclonal Mouse, clone NCH-38, RTU, IR05961) was supplied by Dako; Agilent Technologies, Inc. (Santa Clara, CA, USA). Antigen retrieval was performed using EnVision TM FLEX Target Retrieval Solution High pH (pH 9.0) for 20 min. at 97–98°C in PT Link instrument (Dako; Agilent Technologies, Inc., Santa Clara, CA, USA). Endogenous peroxidase activity was blocked by incubation for 10 min. in 3% hydrogen peroxide, followed by antibody incubation for 20 min. at room temperature. EnVision TM FLEX/HRP, High pH kit (K8000, Dako; Agilent Technologies, Inc., Santa Clara, CA, USA) was used as a detection system according to the manufacturer’s instructions. High E-cadherin expression was classified as homogenous 3+ and heterogeneous 3+ and 2+ staining; low E-cadherin expression was defined in samples with heterogeneous staining covering 1+ and no expression regardless of the portion of cells with 3+, 2+, 1+, and no E-cadherin expression.

### 4.5. Statistical Analysis

IBM SPSS statistics 23.0 software was used for statistical analysis. qPCR data were analyzed using REST 2009 Software (Technical University Munich and Qiagen, Germany). The normality of distribution was assessed by the Shapiro–Wilk test. Normally distributed variables were tested using Student’s t-test. Non-normally distributed data were tested by nonparametric Mann–Whitney U test. Pearson’s or Spearman’s correlations were used to assess the correlations between miRNA and mRNA expression of tested genes. Binary logistic regression was used to evaluate the influence of selected gene and miRNA expression on hematogenous and lymphogenous dissemination of tumor cells and to control for confounders. This determination included enumeration of the risk estimate presented as estimated odds ratio (OR) and 95% confidence interval (CI) for the OR. *p*-value < 0.05 was defined as statistically significant.

## Figures and Tables

**Table 1:** Comparison of miRNA and mRNA of CDH1 and EMT gene expressions between controls and different types of BC patients’ specimens and between each other’s samples.

miRNAs/Genes	TU-C vs. C-Breast			TU-IF vs. C-Breast			LNM vs. C-Breast		
	FC	*p*-Value	95% CI	FC	*p*-Value	95% CI	FC	*p*-Value	95% CI
miR-9-5p	7.915	**0.042**	0.012–41.946	0.838	0.790	0.004–263.197	0.551	0.422	0.004–180.863
miR-124-3p	1.789	0.553	0.001–4.238	1.632	0.557	0.002–1.277	0.649	0.634	0.002–624.915
miR-200c-3p	1.686	0.382	0.054–45.865	2.137	0.092	0.215–59.281	1.821	0.271	0.156–48.471
miR-203a-3p	2.356	**0.042**	0.077–79.574	1.556	0.294	0.042–38.469	1.287	0.593	0.039–29.094
miR-205-5p	0.512	0.251	0.009–36.730	0.350	0.054	0.002–10.925	0.210	**0.012**	0.002–8.057
miR-221-3p	1.335	0.423	0.081–17.503	1.365	0.290	0.168–13.709	1.096	0.802	0.091–19.943
miR-720	1.152	0.735	0.050–33.593	1.398	0.245	0.130–15.221	1.033	0.939	0.042–22.785
*CDH1*	0.123	**0.002**	0.004–2.366	0.108	**0.001**	0.004–2.015	0.630	0.754	0.005–461.981
*TWIST1*	0.449	0.765	0.000–19.953	0.320	0.097	0.000–29.212	2.866	0.717	0.000–84,067.528
*SNAI1*	0.711	0.586	0.027–23.260	0.706	0.658	0.020–55.154	1.460	0.740	0.024–246.709
*SNAI2*	0.161	**0.021**	0.003–13.083	0.102	**0.002**	0.001–9.663	0.302	0.168	0.001–140.598
*ZEB1*	0.482	0.117	0.032–9.616	0.474	0.052	0.064–6.320	22.08	**0.043**	0.083–522,995.072
*ZEB2*	0.125	**<0.001**	0.002–3.578	0.086	**<0.001**	0.001–3.209	0.122	**0.003**	0.001–13.175
	**CTC- vs. C-Blood**			**CTC+ vs. C-Blood**			**CTC+ vs. CTC-**		
	**FC**	***p*-Value**	**95% CI**	**FC**	***p*-Value**	**95% CI**	**FC**	***p*-Value**	**95% CI**
miR-9-5p	0.428	0.366	0.001–157.642	0.374	0.223	0.002–39.997	0.873	0.880	0.004–373.287
miR-124-3p	9.766	**0.036**	0.007–15,744.196	5.914	0.150	0.007–12,237.005	0.606	0.696	0.000–6364.879
miR-200c-3p	0.398	0.171	0.005–43.795	0.299	0.052	0.008–10.359	0.751	0.645	0.012–25.075
miR-203a-3p	1.176	0.643	0.000–745.653	0.148	0.089	0.000–16.253	0.125	0.064	0.000–174.337
miR-205-5p	10.792	0.082	0.004–167,893.014	0.443	0.370	0.002–138.141	0.041	**0.010**	0.000–69.820
miR-221-3p	0.771	0.634	0.023–36.002	0.289	**0.017**	0.016–6.790	0.374	0.077	0.005–14.906
miR-720	0.667	0.451	0.020–37.507	0.534	0.339	0.004–18.729	0.801	0.712	0.006–25.216
*CDH1*	1.031	0.918	0.071–29.950	0.948	0.912	0.132–17.345	0.919	0.782	0.024–7.315
*TWIST1*	0.518	0.362	0.006–18.831	0.685	0.606	0.010–25.111	1.321	0.543	0.052–21.295
*SNAI1*	0.456	0.136	0.016–6.304	0.424	0.125	0.013–7.586	0.931	0.658	0.072–9.000
*ZEB1*	0.683	0.206	0.032–12.101	1.121	0.751	0.029–23.244	1.641	0.410	0.085–19.595
*ZEB2*	0.451	0.066	0.022–6.546	0.395	**0.037**	0.024–2.350	0.876	0.824	0.135–3.364
	**TU-IF vs. TU C**			**LNM vs. TU C**			**LNM vs. TU IF**		
	**FC**	***p*-Value**	**95% CI**	**FC**	***p*-Value**	**95% CI**	**FC**	***p*-Value**	**95% CI**
miR-9-5p	0.106	**0.001**	0.000–48.840	0.070	**<0.001**	0.000–28.042	0.658	0.437	0.005–113.159
miR-124-3p	0.912	0.898	0.000–1595.729	0.363	0.215	0.000–775.000	0.398	0.201	0.001–451.409
miR-200c-3p	1.267	0.531	0.089–30.484	1.080	0.892	0.069–25.098	0.852	0.556	0.064–9.123
miR-203a-3p	0.661	0.199	0.016–16.564	0.546	0.103	0.01 -11.711	0.827	0.614	0.018–32.217
miR-205-5p	0.684	0.612	0.003–68.594	0.409	0.100	0.002–48.151	0.598	0.318	0.005–112.073
miR-221-3p	1.022	0.934	0.079–19.973	0.821	0.540	0.043–18.746	0.803	0.452	0.054–13.541
miR-720	1.213	0.481	0.040–22.891	0.897	0.766	0.015–29.349	0.739	0.325	0.021–11.792
*CDH1*	0.882	0.662	0.048–16.349	5.135	**0.048**	0.081–4310.936	5.819	**0.034**	0.087–4172.813
*TWIST1*	0.712	0.148	0.025–31.480	6.378	0.158	0.017–88,008.904	8.96	0.102	0.019–132,814.422
*SNAI1*	0.994	0.986	0.043–54.895	2.055	0.208	0.052–259.574	2.067	0.224	0.019–316.497
*SNAI2*	0.635	0.198	0.005–16.977	1.875	0.258	0.022–238.470	2.955	0.070	0.025–644.682
*ZEB1*	0.982	0.947	0.111–15.056	45.794	**0.015**	0.139–1,074,172.923	46.615	**0.014**	0.225–1,274,282.570
*ZEB2*	0.69	0.191	0.029–8.390	0.979	0.962	0.027–58.004	1.419	0.415	0.034–76.961

Abbreviations: C-breast, non-neoplastic breast tissue controls; TU-C, tumor centrum; TU-IF, tumor invasive front; LNM, lymph node metastasis; C-blood, CD45-DB fractions from healthy women; CTC- and CTC+, CD45-DB fractions from patients without and with identified epithelial and mesenchymal markers, respectively. Significant results are highlighted in bold.

**Table 2:** Significant correlations between miRNA and mRNA of *CDH1*, and EMT gene expressions in BC samples.

Sample	miRNA	Gene	Correlation Coefficient (r)	*p*-Value
C-breast	miR-124-3p	*SNAI1*	0.964	0.036
TU-C	miR-203a-3p	*CDH1*	0.409	0.031
		*ZEB1*	0.479	0.018
		*ZEB2*	0.406	0.036
	miR-205-5p	*SNAI1*	0.383	0.048
	miR-221-3p	*TWIST1*	**−0.470**	0.015
TU-IF	miR-200c-3p	*SNAI1*	0.387	0.046
	miR-720	*TWIST1*	0.417	0.034
		*SNAI1*	0.527	0.005
		*SNAI2*	0.383	0.040
LNM	miR-9	*SNAI1*	**−0.607**	0.013
	miR-221-3p	*TWIST1*	0.726	0.011
		*ZEB1*	0.581	0.048
C-blood	miR-9	*ZEB1*	0.610	0.046
		*ZEB2*	0.627	0.039
	miR-200c-3p	*ZEB1*	0.616	0.043
		*ZEB2*	0.682	0.021
	miR-203a-3p	*TWIST1*	0.967	0.007
		*SNAI1*	0.797	0.032
		*ZEB1*	0.800	0.010
		*ZEB2*	0.900	0.001
	miR-221-3p	*TWIST1*	0.885	0.019
		*SNAI1*	0.784	0.021
		*ZEB1*	0.785	0.004
		*ZEB2*	0.936	<0.000
	miR-720	*TWIST1*	0.869	0.025
		*ZEB1*	0.743	0.009
		*ZEB2*	0.736	0.010
CTC-	miR-124-3p	*TWIST1*	**−0.883**	0.020
	miR-221-3p	*ZEB2*	**−0.543**	0.024
CTC+	miR-9	*SNAI1*	**−0.835**	0.001
		*ZEB1*	**−0.853**	0.001
	miR-205-5p	*SNAI1*	**−0.653**	0.021
		*ZEB1*	**−0.588**	0.044
	miR-221-3p	*ZEB1*	**−0.610**	0.035
	miR-720	*SNAI1*	**−0.616**	0.033
		*ZEB1*	**−0.623**	0.031

Abbreviations: C-breast, non-neoplastic breast tissue controls; TU-C, tumor centrum, TU-IF, tumor invasive front; LNM, lymph node metastasis; C-blood, CD45-DB fractions from healthy women; CTC- and CTC+, CD45-DB fractions from patients without and with identified epithelial and mesenchymal markers, respectively. Negative correlations are highlighted in bold.

**Table 3:** Significant up- and downregulation of miRNA and mRNA of *CDH1*, and EMT gene expression in BC patients with different clinico-pathological parameters.

Sample	Clinical Characteristics	miRNAs/Genes	FC	*p*-Value	95% CI
TU-C	MTS+ vs. MTS-	miR-124-3p	0.075	0.049	0.000–35.995
	HER2+ vs. HER2-	miR-200c-3p	0.440	0.018	0.030–12.772
	TNM III and IV vs. TNM I and II	miR-203a-3p	0.316	0.008	0.009–4.922
	ER+ vs. ER-	*SNAI1*	0.465	0.007	0.049–7.093
		*ZEB2*	0.486	0.017	0.113–5.252
	PR+ vs. PR-	*CDH1*	0.366	0.025	0.028–3.448
		*SNAI1*	0.336	0.008	0.049–6.071
TU-IF	≤20 mm vs. >20 mm	*ZEB2*	0.343	0.011	0.024–4.821
	LNM+ vs. LNM-	*SNAI2*	0.326	0.023	0.002–9.962
		*ZEB1*	0.497	0.035	0.079–4.408
	MTS+ vs. MTS-	*SNAI2*	0.432	0.041	0.034–94.834
		*ZEB2*	0.344	0.030	0.006–7.949
	TNM III and IV vs. TNM I and II	*SNAI2*	0.246	0.007	0.002–4.248
		*ZEB1*	0.438	0.007	0.059–2.126
	LVI+ vs. LVI-	*SNAI2*	0.294	0.015	0.002–6.602
		*ZEB1*	0.482	0.034	0.079–3.844
		*ZEB2*	0.350	0.026	0.024–3.722
	ER+ vs. ER-	miR-200c-3p	3.795	0.048	0.684–24.343
		*TWIST1*	0.420	0.049	0.011–15.353
LNM	MTS + vs. MTS-	*ZEB1*	824.73	0.018	0.283–1,584,361.881
	TNM III and IV vs. TNM I and II	*SNAI1*	5.959	0.025	0.227–432.565
	ER+ vs. ER-	miR-124-3p	6.819	0.018	0.091–887.828
CD45-DB	CTC+ vs. CTC-	miR-205-5p	0.041	0.010	0.000–69.820
	LNM+ vs. LNM-	miR-205-5p	22.961	0.035	0.012–400,412.929
	TNM III and IV vs. TNM I and II	miR-205-5p	39.056	0.006	0.039–692,990.143
	HER2+ vs. HER2-	miR-9	10.321	0.027	0.088–1098.021
		*SNAI1*	0.439	0.041	0.061–9.288
	Grade 3 vs. Grade 1 and 2	*CDH1*	1.468	0.033	0.151–11.647

Abbreviations: FC, fold change; CI, confidence interval; TU-C, tumor centrum, TU-IF, tumor invasive front; LNM, lymph node metastasis; CD45-DB, CD45-depleted blood; CTC, circulating tumor cell; MTS, metastatic; TNM, TNM staging system (T, tumor; N, lymph node; M, metastasis); LN, lymph node; LVI, lymphovascular invasion; ER, estrogen receptor; PR, progesterone receptor; HER2, human epidermal growth factor receptor 2.

**Table 4:** Risk estimation of miR-205-5p expression and clinical status for the presence of CTC in CD45-DB of BC patients (logistic regression adjusted for age).

Risk Factor	Variables	*p*-Value	OR	95% CI
CTCs presence	Age High grade (G3) miR-205-5p *	0.061 0.075 0.028	0.893 46.197 4.326	0.792–1.005 0.683–3124.868 1.170–15.995
	Tumor size > 20 mm	0.047	96.081	1.066–8661.849
	HER2 positivity ^§^ Constant	0.036 0.126	2153.786 0.000	1.628–2,849,343.631

* ΔCt values in CD45-DB samples; ^§^ cut-off 10%. Abbreviations: CI, Confidence interval; CTCs, circulating tumor cells; HER2, Human epidermal growth factor receptor 2; OR, Odds ratio. Model summary: −2 Log likelihood 18.870; *R* squared (Cox & Snell) 0.512; *R* squared (Nagelkerke) 0.692. Input variables to model: age, histological grade, miR-205-5p expression, tumor size, and HER2 status.

**Table 5:** Clinical characteristics.

Variables		*n*	%
All		31	100.0
Age	≤50	3	9.68
	>50	28	90.32
Histological grade	1 and 2	14	45.16
	3	17	54.84
Tumor size (mm)	≤20	12	38.71
	>20	19	61.29
LNM status ^a^	0	9	29.03
	≥1	22	70.97
MTS status	Negative	24	77.42
	Positive	7	22.58
TNM stage	I. and II.	13	41.94
	III. and IV.	18	58.06
CTC occurrence ^b^	Negative	18	58.06
	Positive	13	41.94
LVI	Negative	7	22.58
	Positive	24	77.42
ER status ^c^	Negative	5	16.13
	Positive	26	83.87
PR status ^c^	Negative	11	35.48
	Positive	20	64.52
HER2 status ^d^	Negative	23	74.19
	Positive	8	25.81
Ki-67 proliferative index ^e^	Low	4	12.90
	High	27	87.10
E-cadherin expression ^f^	High	22	70.97
	Low	9	29.03

Abbreviations: LNM, lymph node metastasis; MTS, metastatic; LVI, lymphovascular invasion; ER, estrogen receptor; PR, progesterone receptor; HER2, human epidermal growth factor receptor 2. ^a^ LNM status was categorized according to the number of metastatic LNs; ^b^ CTC occurrence was evaluated in CD45-depleted blood (CD45-DB) fractions through the absence or presence of epithelial and mesenchymal markers; ^c^ ER, PR was considered as positive in cases with ≥ 1% of positively responding cells; ^d^ HER2 positive cases were those that showed strong homogeneous and circumferential membrane expression in more than 10% of tumor cells (i.e., 3+ intensity) or those that showed a 2+ intensity and subsequent FISH analysis demonstrated amplification of the *HER2* gene. HER2 negative cases were with a response intensity of 0 or 1+, or cases with a response intensity of 2+ without proven amplification; ^e^ Low and high Ki-67 expression according to the number of stained cancer cell with a cut-off 15%; ^f^ High E-cadherin expression was classified as homogenous 3+ and heterogeneous 3+ and 2+ staining; low E-cadherin expression were defined in samples with heterogeneous staining covering 1+ and no expression regardless of portion of cells with 3+, 2+, 1+ and no E-cadherin expression.

## Supplementary Materials

The following are available online at https://www.mdpi.com/article/10.3390/ijms23010103/s1.

## Abbreviations

BC	Breast cancer
C-blood	CD45-depleted blood fraction from healthy women
C-breast	Non-neoplastic breast tissue
CD45-DB	CD45-depleted blood
CDH1	Cadherin 1
CI	Confidence interval
CTC	Circulating tumor cells
EMT	Epithelial-to-mesenchymal transition
ER	Estrogen receptor
FC	Fold change
FFPE	Formalin-fixed paraffin-embedded tissue
HER2	Human epidermal growth factor receptor 2
LN	Lymph node
LNM	Lymph node metastasis
LVI	Lymphovascular invasion
mRNA	Messenger RNA
miRNA	microRNA
MTS	Distant metastasis
OR	Odds ratio
PR	Progesterone receptor
r	Correlation coefficient
RT-PCR	Real time- Polymerase chain reaction
SNAI1	Snail Family Transcriptional Repressor 1
SNAI2	Snail Family Transcriptional Repressor 2
TNBC	Triple-negative breast cancer
TNM	Tumor Node Metastasis staging
TU-C	Tumor centrum
TU-IF	Tumor invasive front
TWIST1	Twist Family BHLH Transcription Factor 1
ZEB1	Zinc Finger E-Box Binding Homeobox 1
ZEB2	Zinc Finger E-Box Binding Homeobox 2
