# Supplementary material for: miR-205-5p Downregulation and ZEB1 Upregulation Characterize the Disseminated Tumor Cells in Patients with Invasive Ductal Breast Cancer
Source: Int J Mol Sci. 2021 Dec 22;23(1):103. doi: 10.3390/ijms23010103 (PMC8744876; doi:10.3390/ijms23010103)
Supplement: Supplementary file 1 [file ijms-23-00103-s001.zip › ijms-1511902-supplementary.pdf]

Table S1. Significant correlations between mRNA expressions of *CDH1* and EMT genes in different types of BC patient's samples

Sample	Gene	Gene	Correlation coefficient (r)	*P*-value
C-breast	*TWIST1*	*SNAI2*	0.710	0.049
		ZEB2	0.830	0.011
	SNAI2	*CDH1*	0.995	0.005
	ZEB1	*SNAI2*	0.818	0.024
	ZEB2	*SNAI2*	0.776	0.005
		ZEB1	0.847	0.016
TU-C	*TWIST1*	*CDH1*	0.468	0.018
	SNAI2	*SNAI1*	0.493	0.009
	ZEB1	*SNAI1*	0.568	0.005
		SNAI2	0.553	0.005
	ZEB2	*SNAI1*	0.587	0.002
		SNAI2	0.775	< 0.000
		ZEB1	0.603	0.002
TU-IF	*SNAI2*	*SNAI1*	0.479	0.013
	ZEB1	*CDH1*	0.430	0.046
		SNAI1	0.500	0.021
		SNAI2	0.672	<0.000
	ZEB2	*TWIST1*	0.426	0.034
		SNAI1	0.431	0.032
		SNAI2	0.579	0.002
		ZEB1	0.462	0.027
LNM	*TWIST1*	*SNAI2*	0.831	0.002
	SNAI2	*CDH1*	0.683	0.007
	ZEB1	*TWIST1*	0.686	0.041
		SNAI1	0.628	0.038
		SNAI2	0.725	0.008
C-blood	*TWIST1*	*SNAI1*	0.958	0.010
	ZEB1	*TWIST1*	0.981	0.001
		SNAI1	0.840	0.009
	ZEB2	*TWIST1*	0.947	0.004
		SNAI1	0.831	0.011
		ZEB1	0.940	< 0.000
CTC-	*ZEB2*	*ZEB1*	0.607	0.010
CTC+	*ZEB1*	*SNAI1*	0.798	0.002

Abbreviations: C-breast, non-neoplastic breast tissue controls; TU-C, tumor central region, TU-IF, tumor invasive front; LNM, lymph node metastasis; C-blood, CD45-depleted blood fractions from healthy women; CTC- and CTC+, CD45-depleted blood fractions from patients without and with identified epithelial and mesenchymal markers, respectively.
